# Predictors of Developing Postoperative Atrial Fibrillation in Patients Undergoing Coronary Artery Bypass Graft: A Systematic Review and Meta-Analysis

**DOI:** 10.7759/cureus.51316

**Published:** 2023-12-30

**Authors:** Martha Mekonen Gdey, Purvi Buch, FNU Pareesa, Mahek Thorani, Hazem Nasser, Revanth Reddy Bandaru, Calvin R Wei, Sujith K Palleti

**Affiliations:** 1 General Practice, Mekelle University, Mekelle, ETH; 2 Medicine, Gujarat Medical Education and Research Society (GMERS) Medical College, Gotri, IND; 3 Medicine, People's University of Medical and Health Sciences Nawabshah, Karachi, PAK; 4 Internal Medicine, People's University of Medical and Health Sciences Nawabshah, Karachi, PAK; 5 Medicine, John H. Stroger, Jr. Hospital of Cook County, Illinois, USA; 6 Internal Medicine, East Carolina University, Greenville, USA; 7 Research and Development, Shing Huei Group, Taipei, TWN; 8 Nephrology, Louisiana State University Health Sciences Center, Shreveport, USA

**Keywords:** systematic review and meta-analysis, post-operative, predictors, atrial fibrillation, coronary artery bypass graft

## Abstract

The objective of this study was to determine predictors of postoperative atrial fibrillation (POAF) among coronary artery bypass graft (CABG) patients. This meta-analysis was conducted as per the guidelines of the Preferred Reporting Items for Systematic Reviews and Meta-analyses (PRISMA). Two authors performed searches independently using electronic databases, including Embase, PubMed, and Web of Science, from January 1, 2015, to November 30, 2023. A total of 16 studies were included in this meta-analysis. All included studies reported POAF in patients undergoing CABG, resulting in 1462 cases of POAF among 6200 patients undergoing CABG. The cases of POAF varied among studies, ranging from 7.80% to 47.37%. The pooled incidence of POAF was 26% (95% CI: 20% to 31%). The results indicated that older patients had a higher risk of developing atrial fibrillation (AF) after CABG (mean difference [MD]): 5.63, 95% confidence interval (CI): 4.08 to 7.17, p-value < 0.001). The findings revealed a significantly lower left ventricular ejection fraction (LVEF) in patients developing AF than their counterparts (MD: −0.30, 95% CI: −0.58 to −0.03, p-value: 0.03). Regarding the history of myocardial infarction (MI), the odds of MI were significantly higher in patients developing AF compared to those who did not develop AF (odds ratio [OR]: 1.37, 95% CI: 1.12 to 1.68, p-value: 0.002). In relation to intra-aortic balloon pump (IABP), the odds of IABP were significantly higher in patients developing AF compared to those who did not develop AF (OR: 2.27, 95% CI: 1.39 to 3.72, p-value: 0.001). Identified risk factors for post-CABG AF included advanced age, a lower preoperative ejection fraction, a history of myocardial infarction, the requirement for an IABP, and prolonged cardiopulmonary bypass (CPB) time. The study underscores the significance of proactive screening and comprehensive management for elderly CABG patients, particularly those with myocardial infarction histories.

## Introduction and background

Atrial fibrillation (AF) is a commonly observed complication subsequent to cardiac surgery, occurring at a reported frequency of 10 to 50%, depending on various definitions and diagnostic methods [1]. Typically manifesting between two and four days post-surgery, AF frequently recurs within the initial 30 days of the postoperative period [2]. Recent observations indicate an increased incidence of AF, likely linked to the expansion of the patient pool undergoing coronary artery bypass grafting (CABG) or other open-heart procedures due to a reduction in contraindications [3].

The consequences of postoperative atrial fibrillation (POAF) encompass both pathophysiological and financial implications. Numerous studies have demonstrated a twofold rise in morbidity and mortality rates, encompassing both all-cause 30-day and six-month mortality. Postoperative AF is correlated with heightened patient mortality, prolonged hospitalization, hemodynamic complications, and thromboembolism [4]. POAF appears to arise when transient postoperative triggers impact vulnerable atrial tissue, influenced by preoperative, procedure-induced, and postoperative arrhythmogenic processes such as inflammation, oxidative stress, autonomic dysfunction, and electrophysiological remodeling [5]. Understanding its pathogenesis is crucial for identifying preventive and therapeutic measures to enhance POAF outcomes post-cardiac surgery.

Effective management and prevention of postoperative AF are vital for enhancing patients' health, expediting rehabilitation, and minimizing hospitalization costs. Despite guidelines from reputable organizations such as the American Heart Association, European Society of Cardiology, and American College of Cardiology, uncertainties persist regarding the choice of antiarrhythmic drugs, timing and duration of therapy, and prevention of recurrences [6-7]. Recognizing patients at risk of new-onset POAF after CABG is clinically significant for implementing appropriate precautions during the perioperative period to optimize clinical results.

Previously identified predisposing factors for POAF after CABG include advanced age, obesity, and comorbidities such as hypertension, diabetes mellitus, and chronic obstructive pulmonary disease (COPD) [8-9]. However, conclusive risk factors for new-onset POAF after CABG remain elusive. Thus, our objective is to determine predictors of POAF among CABG patients through a systematic review of available research evidence.

## Review

Methods

This meta-analysis was conducted as per the guidelines of the Preferred Reporting Items for Systematic Reviews and Meta-analyses (PRISMA).

Search Strategy and Literature Search

Two authors performed searches independently using electronic databases, including Embase, PubMed, and Web of Science, from January 1, 2015, to November 30, 2023. Besides this, searching was also done in Google Scholar to find additional studies relevant to the study topic. We identified relevant articles from the references to these articles and removed duplicates. The search strategy included the following terms: “coronary artery bypass grafting," “atrial fibrillation," and "predictors," along with synonyms and medical subject heading (MeSH) terms.

Study Selection

After the initial search, two authors independently screened all titles and abstracts and resolved discrepancies through consultation with a third author. The studies that met the following criteria were included in this meta-analysis: (i) full-text; (ii) patients with an age of 18 years or more; and (3) CABG performed for the treatment of coronary artery disease (CAD). This meta-analysis included prospective and retrospective studies assessing the risk factors for new-onset POAF after CABG. We excluded reviews, letters to the editor, editorials, reviews, and commentaries. We excluded studies that underwent surgery other than CABG and studies from which we were unable to extract unadjusted data related to risk factors. Lastly, we excluded studies assessing the effectiveness of any intervention.

Data Extraction and Quality Assessment

Two authors conducted independent data extraction and quality assessment, cross-verifying the extracted information to achieve consensus. The extracted data encompassed author names, publication year, study type, study region, sample size, the number of patients developing atrial fibrillation post-CABG, and associated risk factors. The quality assessment of selected studies utilized the Newcastle-Ottawa Scale (NOS), an eight-item scale evaluating three domains: selection, comparability, and outcome. The maximum score of 9 is distributed into 4 points for selection, 2 for comparability, and 3 for outcome. Total scores are interpreted as follows: 7-9 for good quality, 4-6 for fair quality, and 0-3 for poor quality. The NOS was independently scored by two authors, with conflicts resolved through discussion.

Statistical Analysis

The primary focus was on identifying the risk factors associated with postoperative AF following CABG. To achieve this, we conducted a comparison of demographic and clinical characteristics between patients who developed AF and those who did not after CABG. The meta-analysis results were presented as pooled odds ratios (ORs) with 95% confidence intervals (CIs) for categorical variables and pooled mean differences (MD) with 95% CI for continuous variables. RevMan version 5.4.1 (The Nordic Cochrane Centre, The Cochrane Collaboration, Copenhagen) was employed for the meta-analysis, with statistical significance set at a two-sided P-value < 0.05. Statistical heterogeneity was assessed using the I2 statistic. The random effect model was used irrespective of the heterogeneity to deal with the variation among the studies.

Results

**Figure 1:**
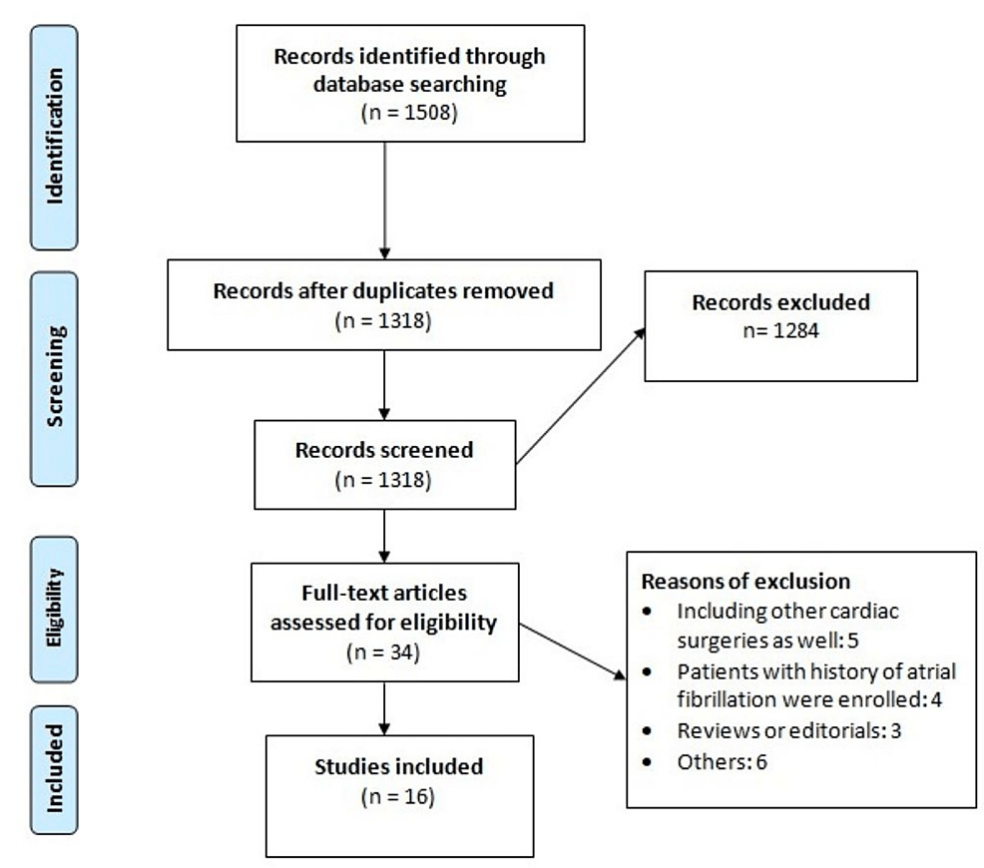
PRISMA flowchart of study selection

A total of 1508 studies were initially obtained after online database searching. After removing duplicate studies, 1318 records remained for initial screening using titles and abstracts. Of these studies, 34 were selected for full-text screening. A total of 16 studies were ultimately included in this meta-analysis. Details of the PRISMA chart for study selection are shown in Figure 1. Table 1 shows the characteristics of the included studies. Out of the 17 included studies, 7 were prospective. Table 2 presents the quality assessment of the included studies.

**Table 1:** Characteristics of included studies

Author name	Year	Region	Study design	Sample size (N)	Atrial fibrillation (n)
Choi et al. [10]	2021	South Korea	Retrospective	327	93
Daie et al. [11]	2018	Iran	Prospective	156	29
El-Gendy et al. [12]	2020	Egypt	Prospective	123	41
Erdil et al. [13]	2022	Turkey	Retrospective	490	62
Folla et al. [14]	2016	Portugal	Retrospective	105	20
Ismail et al. [15]	2017	Saudi Arabia	Retrospective	252	84
Junior et al. [16]	2015	Brazil	Retrospective	134	18
Lee et al. [17]	2018	South Korea	Retrospective	999	244
Lotter et al. [18]	2023	Australia	Retrospective	388	98
Musa et al. [19]	2018	Malaysia	Retrospective	637	183
Omar et al. [20]	2021	Egypt	Prospective	1000	78
Rajabi et al. [21]	2020	Iran	Retrospective	261	85
Rubanenko et al. [22]	2023	Russia	Prospective	158	47
Tsai et al. [23]	2015	Taiwan	Prospective	266	126
Velioglu [24]	2019	Turkey	Prospective	458	143
Vlahou [25]	2016	Greece	Prospective	446	111

**Table 2:** Quality assessment of included studies

Author Name	Selection	Comparison	Outcome	Overall
Choi et al [10]	3	2	3	Good
Daie et al [11]	4	2	2	Good
El-Gendy et al [12]	3	2	3	Good
Erdil et al [13]	2	2	2	Good
Folla et al [14]	3	1	2	Fair
Ismail et al [15]	4	2	3	Good
Junior et al [16]	3	2	2	Good
Lee et al [17]	4	2	3	Good
Lotter et al [18]	4	1	3	Good
Musa et al [19]	4	1	3	Good
Omar et al [20]	3	2	2	Good
Rajabi et al [21]	2	2	3	Good
Rubanenko et al [22]	4	1	2	Good
Tsai et al [23]	3	2	2	Good
Velioglu [24]	3	1	2	Fair
Vlahou [25]	3	2	3	Good

Incidence of Postoperative Atrial Fibrillation in Patients Undergoing CABG

All included studies reported postoperative AF in patients undergoing CABG, resulting in 1462 cases of postoperative AF among 6200 patients undergoing CABG. The cases of postoperative AF varied among studies, ranging from 7.80% to 47.37%. The pooled incidence of postoperative atrial fibrillation was 26% (95% CI: 20% to 31%).

*Risk Factors for the Development of Atrial Fibrillation in Patients Undergoing CABG* 

All studies included in this analysis provided detailed information on patients who either developed AF or did not follow CABG. Given the diverse clinical and demographic characteristics reported across these studies, a thorough examination of the corresponding data was conducted. Data reported by fewer than three studies were deemed insufficient and subsequently excluded. The final analysis considered the following variables: age, gender, smoking history, body mass index (BMI), diabetes mellitus, hypertension, dyslipidemia, COPD, history of myocardial infarction (MI), cardiopulmonary bypass time (CPB), intra-aortic balloon pump (IABP), left ventricular ejection fraction (LVEF), and preoperative use of drugs (beta-blockers, angiotensin-converting enzyme [ACE] inhibitors, statins, and aspirin). These variables are summarized in Tables 3-4. Table 3 shows the relationship of POAF with categorical variables, while Table 4 presents continuous variables.

**Table 3:** Factors associated with development of postoperative atrial fibrillation (categorical variables) OR: odds ratio; CI: confidence interval; MI: myocardial infarction; COPD: chronic obstructive pulmonary disease; IABP: intra-aortic balloon pump; ACE: angiotensin-converting enzyme

Variable	Number of studies	OR (95% CI)	P-value	I-square
Gender (male)	14	0.82 (0.66–1.03)	0.08	50%
Smoking	10	0.81 (0.63–1.03)	0.08	50%
Diabetes	13	1.09 (0.88–1.34)	0.43	54%
Hypertension	12	1.11 (0.88–1.39)	0.38	55%
Dyslipidemia	9	1.05 (0.88–1.26)	0.6	0%
Previous MI	6	1.37 (1.12–1.68)	0.002	0%
COPD	5	1.37 (0.63–2.98)	0.43	72%
IABP	5	2.27 (1.39–3.72)	0.001	0%
Beta-blockers	10	0.76 (0.52–1.12)	0.17	76%
ACE Inhibitors	4	1.20 (0.93–1.56)	0.23	31%
Statin	6	0.84 (0.65–1.09)	0.36	8%
Aspirin	4	0.80 (0.64–1.01)	0.06	0%

**Table 4:** Factors associated with postoperative atrial fibrillation (continuous variables) MD: mean difference; CI: confidence interval; BMI: body mass index; LVEF: left ventricular ejection fraction; CPB: cardiopulmonary bypass

Variable	Number of studies	MD (95% CI)	P-value	I-square
Age	13	5.63 (4.08 to 7.17)	0.001	86%
LVEF	12	−0.30 (-0.58 to -0.03)	0.03	94%
BMI	8	−0.50 (−1.20 to 0.21)	0.17	70%
CPB time	5	4.81 (0.43 to 9.19)	0.03	28%

Thirteen studies provided data on the age of patients who developed AF and those who did not follow CABG. The results indicated that older patients had a higher risk of developing AF after CABG (MD: 5.63, 95% CI: 4.08 to 7.17, p-value < 0.001). Seven studies compared the preoperative LVEF between the two groups. The findings revealed a significantly lower LVEF in patients developing AF compared to their counterparts (MD: −0.30, 95% CI: −0.58 to −0.03, p-value: 0.03). Regarding the history of MI, the odds of MI were significantly higher in patients developing AF compared to those who did not develop AF (OR: 1.37, 95% CI: 1.12 to 1.68, p-value: 0.002). In relation to IABP, the odds of IABP were significantly higher in patients developing AF compared to those who did not develop AF (OR: 2.27, 95% CI: 1.39 to 3.72, p-value: 0.001). However, the analysis revealed that gender, smoking, diabetes mellitus, hypertension, dyslipidemia, COPD, body mass index (BMI), and preoperative use of drugs (beta-blockers, ACE inhibitors, statins, and aspirin) were not associated with an increased risk of developing postoperative AF.

Discussion

The present meta-analysis assesses the factors associated with the development of postoperative AF after CABG. The pooled incidence of postoperative AF was 26% (95% CI: 20% to 31%). The incidence of postoperative AF in individual studies ranged from 7.80% to 47.37% and the differences in the incidence of postoperative AF are due to study setting, population characteristics, and inclusion and exclusion criteria. The risk factors identified in our meta-analysis affecting the development of postoperative AF included age, ejection fraction, previous history of myocardial infarction, requirement of IABP, and CPB time.

Age emerged as a significant predictor of postoperative AF following CABG. Advanced age stands out as one of the most commonly acknowledged risk factors for the development of AF after CABG. The heightened incidence of AF in older individuals may be linked to age-related comorbidities [26]. This association has traditionally been attributed to aging-induced degenerative alterations in the atrium and modifications in atrial physiology. Amar et al. characterized these changes as involving 'shorter effective refractoriness, delayed SA and AV nodal conduction times, atrial stiffening, and splitting of the atrial excitation waveform caused by the pectinated trabeculae' [27]. In the contemporary landscape of cardiac surgery, where more than half of the procedures are conducted on patients aged 75 and above, it is imperative to implement proactive screening and enhance pre-, peri-, and postoperative management for elderly individuals undergoing CABG [28-29].

The current meta-analysis revealed that patients who later developed AF after CABG had a significantly lower preoperative mean ejection fraction compared to those who did not. Kannel et al. documented an elevated incidence of atrial fibrillation in the general population with diminished left ventricular contractility [30]. Our study corroborates this finding, indicating that individuals with a history of myocardial infarction face an increased risk of postoperative AF. Previous research has consistently shown that patients with prior ischemic events or a myocardial infarction history exhibit a higher susceptibility to developing postoperative AF, potentially attributable to compromised left ventricular function, enlarged dimensions, and myocardial ischemia [31].

The collective outcomes of the meta-analysis indicated a potential association between extended CPB duration and the emergence of new-onset POAF following CABG. These results align with the findings from a meta-analysis by Seo et al., which encompassed nine prospective cohort studies [32]. However, the impact of CPB utilization on the incidence of POAF after CABG warrants additional scrutiny. A comprehensive understanding of CPB application in CABG is crucial, given that POAF is not only a transient complication but also carries long-term consequences in terms of both mortality and the risk of stroke [33-34]. Consequently, further research is imperative to facilitate meta-analyses encompassing variables such as intra- and postoperative risk factors for the onset of new POAF after CABG.

While the precise mechanism and resolution for POAF remain elusive, this complication imposes a substantial burden on patients, hospitals, and families. According to a study, patients developing POAF after CABG face a twofold increase in mortality risk (p < 0.01) [35]. Beyond its association with elevated mortality, POAF also generates a considerable financial strain on patients and their families. Findings from a randomized control trial revealed a one-year cost difference of +$15,593 USD for patients experiencing POAF, attributed to extended hospital stays and complications associated with POAF [36].

This study has several limitations that should be acknowledged when interpreting the results. First, data measured in intensive care units, such as postoperative vital signs, could not be systematically included in our analysis. Additionally, important cardiac-related variables, such as the E-to-A ratio, cardiac index, stroke volume, and cardiac output, were not assessed. Future studies should prioritize the examination of these parameters to better understand their potential impact on the development of atrial fibrillation following CABG.

## Conclusions

In conclusion, our meta-analysis, comprising 17 studies involving 6200 patients undergoing CABG, revealed a pooled incidence of postoperative AF at 26% (95% CI: 20% to 31%). Identified risk factors for post-CABG AF included advanced age, a lower preoperative ejection fraction, a history of myocardial infarction, the requirement for an IABP, and prolonged CPB time. The study underscores the significance of proactive screening and comprehensive management for elderly CABG patients, particularly those with myocardial infarction histories. While longer CPB time is correlated with new-onset post-CABG AF, further research is essential to elucidate intra- and postoperative risk factors. Importantly, post-CABG AF not only doubles mortality risk but also imposes a considerable financial burden on patients and families, emphasizing the need for effective preventive strategies.

## References

[1] Creswell LL, Damiano RJ Jr (2001). Postoperative atrial fibrillation: an old problem crying for new solutions. J Thorac Cardiovasc Surg.

[2] Banach M, Okoński P, Rysz J, Irzmański R, Piechowiak M, Zasłonka J (2005). Atrial fibrillation following cardiosurgical operations - current guidelines of pharmacotherapy and invasive treatment. Pol J Surg.

[3] Healey JS, McIntyre WF (2019). The RACE to treat atrial fibrillation in the emergency department. N Engl J Med.

[4] Shen J, Lall S, Zheng V, Buckley P, Damiano RJ Jr, Schuessler RB (2011). The persistent problem of new-onset postoperative atrial fibrillation: a single-institution experience over two decades. J Thorac Cardiovasc Surg.

[5] Rezaei Y, Peighambari MM, Naghshbandi S (2020). Postoperative atrial fibrillation following cardiac surgery: from pathogenesis to potential therapies. Am J Cardiovasc Drugs.

[6] Fuster V, Rydén LE, Asinger RW (2001). ACC/AHA/ESC guidelines for the management of patients with atrial fibrillation. A report of the American College of Cardiology/American Heart Association Task Force on Practice Guidelines and the European Society of Cardiology Committee for Practice Guidelines and Policy Conferences (Committee to develop guidelines for the management of patients with atrial fibrillation) developed in collaboration with the North American Society of Pacing and Electrophysiology. Eur Heart J.

[7] Okonski P, Szymanska E, Jaszewski R (2004). Lodz surgical risk scale: practical use of the statistical analysis of the risk related to the surgical treatment of coronary artery disease. Pol J Surg.

[8] Burrage PS, Low YH, Campbell NG, O'Brien B (2019). New-onset atrial fibrillation in adult patients after cardiac surgery. Curr Anesthesiol Rep.

[9] Perrier S, Meyer N, Hoang Minh T (2017). Predictors of atrial fibrillation after coronary artery bypass grafting: a Bayesian analysis. Ann Thorac Surg.

[10] Choi HJ, Seo EJ, Choi JS, Oh SJ, Son YJ (2022). Perioperative risk factors for new-onset postoperative atrial fibrillation among patients after isolated coronary artery bypass grafting: A retrospective study. J Adv Nurs.

[11] Daie M, Hajhossein Talasaz A, Karimi A, Gholami K, Salehiomran A, Ariannejad H, Jalali A (2018). Relationship between vitamin D levels and the incidence of post coronary artery bypass graft surgery atrial fibrillation. J Tehran Heart Cent.

[12] El-Gendy HA, Dabsha MH, Elewa GM, Ali AH (2020). Predictors of postoperative atrial fibrillation after coronary artery bypass grafting: a prospective observational cohort study. Ain-Shams J Anesthesiol.

[13] Erdil N, Akca B (2022). Risk factors of postoperative atrial fibrillation in patients undergoing beating heart coronary artery bypass. AZJCVS.

[14] Folla CO, Melo CC, Silva RC (2016). Predictive factors of atrial fibrillation after coronary artery bypass grafting. Einstein (Sao Paulo).

[15] Ismail MF, El-Mahrouk AF, Hamouda TH, Radwan H, Haneef A, Jamjoom AA (2017). Factors influencing postoperative atrial fibrillation in patients undergoing on-pump coronary artery bypass grafting, single center experience. J Cardiothorac Surg.

[16] Bohatch Júnior MS, Matkovski PD, Di Giovanni FJ, Fenili R, Varella EL, Dietrich A (2015). Incidence of postoperative atrial fibrillation in patients undergoing on-pump and off-pump coronary artery bypass grafting. Rev Bras Cir Cardiovasc.

[17] Lee J, Jang I (2020). Predictors affecting postoperative atrial fibrillation in patients after coronary artery bypass graft. Clin Nurs Res.

[18] Lotter K, Yadav S, Saxena P, Vangaveti V, John B (2023). Predictors of atrial fibrillation post coronary artery bypass graft surgery: new scoring system. Open Heart.

[19] Farouk Musa A, Quan CZ, Xin LZ, Soni T, Dillon J, Hay YK, Nordin RB (2018). A retrospective study on atrial fibrillation after coronary artery bypass grafting surgery at the National Heart Institute, Kuala Lumpur. F1000Res.

[20] Omar A, Elshihy EM, Singer M, Zarif D, Dawoud O (2021). Perioperative Risk Factors Predisposing to Atrial Fibrillation After CABG Surgery. Heart Surg Forum.

[21] Rajabi M, Borzou SR, Moeinipour A, Hoseinikhah H, Safarpoor G (2020). Effects of preoperative risk factors on the occurrence of atrial fibrillation following coronary artery bypass in farshchian cardiovascular subspecialty hospital. Iranian Heart J.

[22] Rubanenko O, Rubanenko A, Davydkin I (2023). Comprehensive analysis of factors associated with new episode of postoperative atrial fibrillation after coronary artery bypass graft surgery. Life (Basel).

[23] Tsai YT, Lai CH, Loh SH (2015). Assessment of the risk factors and outcomes for postoperative atrial fibrillation patients undergoing isolated coronary artery bypass grafting. Acta Cardiol Sin.

[24] Velioglu Y, Yuksel A (2019). Predictors of postoperative atrial fibrillation after beating-heart coronary artery bypass surgery: is cardiopulmonary bypass a risk factor?. Acta Cardiol Sin.

[25] Vlahou A, Diplaris K, Ampatzidou F, Karagounnis L, Drossos G (2016). The role of blood transfusion in the development of atrial fibrillation after coronary artery bypass grafting. Thorac Cardiovasc Surg.

[26] Aranki SF, Shaw DP, Adams DH (1996). Predictors of atrial fibrillation after coronary artery surgery. Current trends and impact on hospital resources. Circulation.

[27] Amar D, Zhang H, Leung DH, Roistacher N, Kadish AH (2002). Older age is the strongest predictor of postoperative atrial fibrillation. Anesthesiology.

[28] Knapik P, Hirnle G, Kowalczuk-Wieteska A (2020). Off-pump versus on-pump coronary artery surgery in octogenarians (from the KROK Registry). PLoS One.

[29] Morici N, De Rosa R, Crimi G (2020). Characteristics and outcome of patients ≥75 years of age with prior coronary artery bypass grafting admitted for an acute coronary syndrome. Am J Cardiol.

[30] Kannel WB, Abbott RD, Savage DD, McNamara PM (1982). Epidemiologic features of chronic atrial fibrillation: the Framingham study. N Engl J Med.

[31] Creswell LL, Schuessler RB, Rosenbloom M, Cox JL (1993). Hazards of postoperative atrial arrhythmias. Ann Thorac Surg.

[32] Seo EJ, Hong J, Lee HJ, Son YJ (2021). Perioperative risk factors for new-onset postoperative atrial fibrillation after coronary artery bypass grafting: a systematic review. BMC Cardiovasc Disord.

[33] Kerwin M, Saado J, Pan J, Ailawadi G, Mazimba S, Salerno M, Mehta N (2020). New-onset atrial fibrillation and outcomes following isolated coronary artery bypass surgery: a systematic review and meta-analysis. Clin Cardiol.

[34] Jawitz OK, Gulack BC, Brennan JM (2020). Association of postoperative complications and outcomes following coronary artery bypass grafting. Am Heart J.

[35] Bae SJ, Kwon CH, Kim TY, Chang H, Kim BS, Kim SH, Kim HJ (2022). Predictors and prognostic impact of post-operative atrial fibrillation in patients with hip fracture surgery. World J Clin Cases.

[36] Almassi GH (2015). Postoperative atrial fibrillation; the search goes on. J Surg Res.

